# State of Charge Estimation of Lithium-Ion Batteries Based on an Adaptive Iterative Extended Kalman Filter for AUVs

**DOI:** 10.3390/s22239277

**Published:** 2022-11-29

**Authors:** You Fu, Binhao Zhai, Zhuoqun Shi, Jun Liang, Zhouhua Peng

**Affiliations:** School of Marine Electrical Engineering, Dalian Maritime University, Dalian 116024, China

**Keywords:** lithium-ion battery, state of charge, forgetting factor recursive least squares, adaptive iterative extended Kalman filter

## Abstract

As a power source for autonomous underwater vehicles (AUVs), lithium-ion batteries play an important role in ensuring AUVs’ electric power propulsion performance. An accurate state of charge (SOC) estimation method is the key to achieving energy optimization for lithium-ion batteries. Due to the complicated ocean environments, traditional filtering methods cannot effectively estimate the SOC of lithium-ion batteries in an AUV. Based on the standard extended Kalman filter (EKF), an adaptive iterative extended Kalman filter (AIEKF) method for the SOC in an AUV is proposed to address the traditional filter’s problems, such as low accuracy and large errors. In this method, the adaptive update is introduced to deal with the uncertain noise from the lithium-ion battery. The iteration is used to improve the convergence speed and to reduce the computational burden. Compared with the EKF, iterative extended Kalman filter (IEKF) and adaptive extended Kalman filter (AEKF), the proposed AIEKF has a higher estimation accuracy and anti-interference capability, which is suitable for the AUV’s SOC estimation. In addition, based on the second-order equivalent circuit model of the lithium-ion battery, a forgetting factor recursive least squares (FFRLS) method is proposed to deal with the multi-variability problem. In the end, four different methods, including EKF, IEKF, AEKF, and the proposed AIEKF, are compared in computational time. The experiment results show that the proposed method has high accuracy and fast estimation speed, meaning that it has good application potential in AUVs.

## 1. Introduction

AUVs are multi-functional underwater vehicles capable of autonomous propulsion. A battery provides the power source for an AUV’s propulsion, its range, and the development of various technologies. Therefore, it is critical to select a high-performance underwater power battery technology for AUVs. Lithium-ion batteries with a low self-discharge rates, high energy ratios, and long cycle life are reliable solutions for AUV energy storage. The SOC of lithium-ion batteries reflects the power consumption condition and remaining capacity of the battery, and SOC estimation is an important function of the battery management system (BMS) [1,2,3]. Because SOC cannot be measured directly in practical applications, a high-precision estimation method of SOC is essential to more effectively manage lithium-ion batteries and achieve optimal control of the charging and discharging process [4,5]. Additionally, the state of health (SOH) is another important factor to measure the ability of lithium-ion batteries to safely operate, and the deterioration of the battery will have an impact on the SOC estimation of lithium-ion batteries; therefore, it is crucial to accurately predict the health of the battery [6].

At present, many methods for SOC estimation have been proposed. The ampere-hour counting method [7] is simple to implement, but the initial value of the SOC and the current measurement noise can affect the estimation accuracy, which can lead to a significant increase in the estimation error after accumulation. The open-circuit voltage (OCV) method [8] is used to estimate SOC by determining the function between OCV and SOC, and then the SOC is estimated from the OCV value, but this method requires the battery to sit for sufficient time to reach internal equilibrium, so it is not suitable for online estimation. In addition, because the OCV–SOC curve of lithium-ion batteries is flat in the middle part, a slight deviation of OCV may lead to a large error in SOC estimation. The data-driven approach enables the direct use of data for battery SOC estimation, such as neural networks [9], support vector machines [10], fuzzy systems [11], and so on. However, data-driven methods require a large amount of data for simulation training and the performance depends on the quality of the training dataset. Poor data allocation can also affect the estimation results. Accurate estimation of battery SOC and parameter identification cannot be achieved without modeling it. Equivalent circuit models have the advantage of being simple and easy to implement online, and have been widely used in battery SOC estimation. An accurate and efficient equivalent circuit model is an important factor for effective SOC estimation. The commonly used equivalent circuit models are the Rint model, Thevenin model, and multi-order dynamic model [12,13,14]. Meng et al. [15] proposed an extended battery equivalent circuit model and performed an observability analysis of the nonlinear extended model, which provides important theoretical support for the battery charging control design and the development of a battery monitoring framework. A commonly used model-based approach is to combine the equivalent circuit model with a filter to estimate the SOC. They generally include the extended Kalman filter [16], unscented Kalman filter [17], cubature Kalman filter [18], H∞ filter [19], particle filter [20], and so on. Misyris et al. proposed a hybrid SOC estimation technique in [21], which combines the advantages of three different SOC estimation methods and can effectively improve the estimation accuracy under different conditions, reducing the computational burden and obtaining more accurate SOC estimates. Before the battery SOC estimation, the parameters of the battery model must be identified, and the parameter accuracy of the battery model affects the estimation performance of the battery equivalent circuit model. The commonly used parameter identification methods are the offline identification method [22], recursive least squares method [23,24,25], genetic algorithm [26,27], and particle swarm optimization method [28].

In order to overcome the shortcomings of the traditional EKF in terms of SOC estimation accuracy, an iterative part is added to the EKF. The IEKF updates the Jacobi matrix of the observation equation by bringing the obtained a posteriori estimates into the observation equation, and in each iteration of the calculation process. The estimated value is continuously kept close to the true value by continuously using the observed value during each iteration of the calculation. The absolute value of the difference between the estimated terminal voltage and the measured terminal voltage is set as the threshold value to determine whether to perform the iteration or not. When the voltage error value is less than the threshold value, this process meets the accuracy requirements, and the iteration process is not required. This avoids unnecessary iterative processes and reduces the computational burden. When the voltage error is greater than the threshold value, an iterative process is required. The uncertainty due to system noise affects the accuracy of SOC estimation. To solve this problem, we incorporated the adaptive update scheme. An improved Sage–Husa estimator was used for adaptive updating of process noise and measurement noise, which overcomes the fluctuation of noise due to the influence of external factors. Based on the above, this paper proposes the adaptive iterative extended Kalman filter.

The rest of the paper is structured as follows. Section 2 describes the structure of the battery model chosen in this paper and the FFRLS parameter identification method. Section 3 presents the principle of SOC estimation based on AIEKF. Section 4 conducts experiments based on LiFePO_4_ batteries to verify the reliability of the proposed method. Finally, Section 5 presents the conclusions of this paper.

## 2. Lithium-Ion Battery Modeling

### 2.1. Equivalent Circuit Model

**Figure 1:**
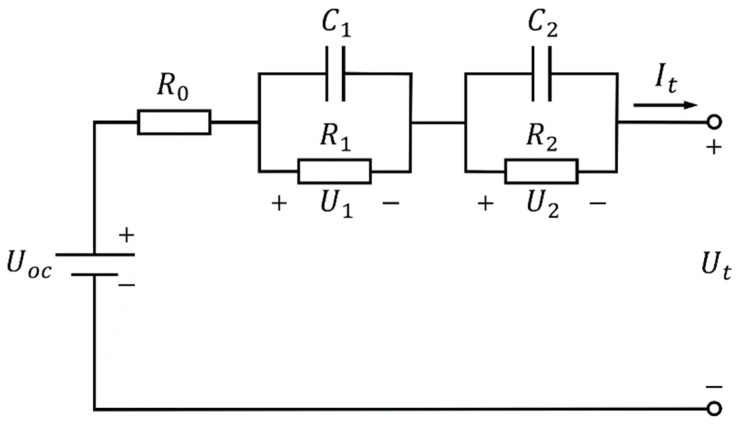
Second-order equivalent circuit diagram of lithium-ion battery. where $U_{oc}$ is the battery’s open-circuit voltage; $U_{1}$ is the battery’s electrochemical polarization voltage; $U_{2}$ is the battery’s concentration polarization voltage; $U_{t}$ is the terminal voltage of the battery; $I_{t}$ is the operating current of the battery; $R_{0}$ is the battery’s ohmic resistance; $R_{1}$ and $C_{1}$ are the battery’s electrochemical polarization resistance and polarization capacitance, respectively, characterizing the slow electrode reaction inside the battery; and $R_{2}$ and $C_{2}$ are the battery’s concentration polarization resistance and polarization capacitance, respectively, characterizing the fast electrode reaction inside the battery.

The second-order equivalent circuit model is more accurate than the first-order equivalent circuit model because increasing the number of RC networks can improve the accuracy of the model. The second-order equivalent circuit model has less computational complexity than other higher-order circuit models and better reflects its polarization characteristics [29]. Therefore, in the case of low system excitation [21], the second-order equivalent circuit model was chosen for the SOC estimation of lithium batteries. The structure of the second-order RC equivalent circuit model is shown in Figure 1.

The second-order equivalent circuit model equation can be derived from Figure 1:

(1) $$\{\begin{array}{c} I_{t}=C_{1}\frac{dU_{1}}{dt}+\frac{U_{1}}{R_{1}}\\ I_{t}=C_{2}\frac{dU_{2}}{dt}+\frac{U_{2}}{R_{2}}\\ U_{t}=U_{oc}-U_{1}-U_{2}-I_{t}R_{0} \end{array}$$

SOC reflects the remaining capacity of the battery and is defined as the ratio of the remaining capacity of the battery to the total capacity with the following formula:

(2) $$SOC_{t}=SOC_{t_{0}}-\int_{t_{0}}^{t}\frac{\eta I_{t}}{Q_{c}}dt$$

where $SOC_{t}$ denotes the SOC at time t, $SOC_{t_{0}}$ denotes the SOC at time t, η is the Coulomb efficiency coefficient, and $Q_{c}$ is the nominal capacity of the battery.

According to the second-order RC equivalent circuit model of the lithium-ion battery, the current is taken as the model input, and the voltage $U_{t}$ is taken as the model output.

Select ${\left[U_{1},U_{2},SOC\right]}^{Τ}$ as the state variable to establish the state space equation of the battery:

(3) $$\left[\begin{array}{c} U_{1,k+1}\\ U_{2,k+1}\\ SOC_{k+1} \end{array}\right]=\left[\begin{array}{ccc} e^{-\frac{t}{\tau_{1}}} & 0 & 0\\ 0 & e^{-\frac{t}{\tau_{2}}} & 0\\ 0 & 0 & 1 \end{array}\right]\left[\begin{array}{c} U_{1,k}\\ U_{2,k}\\ SOC_{k} \end{array}\right]+\left[\begin{array}{c} R_{1}\left(1-e^{-\frac{t}{\tau_{1}}}\right)\\ R_{2}\left(1-e^{-\frac{t}{\tau_{2}}}\right)\\ -\frac{\eta I_{t}}{Q_{c}} \end{array}\right]I_{t,k}+\left[\begin{array}{c} w_{1,k}\\ w_{2,k}\\ w_{3,k} \end{array}\right]$$

(4) $$U_{t,k}=U_{oc}\left(SOC_{k}\right)-U_{1,k}-U_{2,k}-v_{k}$$

where $\tau_{1}=R_{1}C_{1}$, $\tau_{2}=R_{2}C_{2}$, the state variable is $x_{k}={\left[U_{1,k},U_{2,k},SOC_{k}\right]}^{Τ}$, the control variable is $u_{k}=I_{t,k}$, the observation variable is $y_{k}=U_{t,k}$, and the system noise is $w_{k}={\left[w_{1,k},w_{2,k},w_{3,k}\right]}^{Τ}$, whose covariance is Q, and the observation noise is $v_{k}$, whose covariance is R.

### 2.2. Parameter Identification of the Battery Model

In order to establish an accurate battery model, multiple unknown parameters in the model need to be identified. In this study, an online parameter identification method based on the FFRLS algorithm was used to reduce the influence of historical data on new data during the recursive process by setting the forgetting factor and continuously updating the model parameters to obtain more accurate battery parameters.

The Laplace equation of the battery model is obtained from Equation (1).

(5) $$G\left(s\right)=\frac{U_{oc}\left(s\right)-U_{t}\left(s\right)}{I\left(s\right)}=\frac{U\left(s\right)}{I\left(s\right)}=R_{0}+\frac{R_{1}}{1+\tau_{1}s}+\frac{R_{2}}{1+\tau_{2}s}$$

The above equation is discretized using a bilinear variation, so that $s=\frac{2}{T}\frac{1-z^{-1}}{1+z^{-1}}$, and the discretized transfer function can be expressed as:

(6) $$G\left(z^{-1}\right)=\frac{b_{3}+b_{4}z^{-1}+b_{5}z^{-2}}{1-b_{1}z^{-1}+b_{2}z^{-2}}$$

where z is the discrete operator, T is the sampling time, and $b_{1},b_{2},b_{3},b_{4},b_{5}$ are the coefficients to be determined.

Equation (1) can be rewritten as a difference equation.

(7) $$U\left(k\right)=b_{1}U\left(k-1\right)+b_{2}U\left(k-2\right)+b_{3}I\left(k\right)+b_{4}I\left(k-1\right)+b_{5}I\left(k-2\right)$$

Let ψ(k)=[U(k−1) U(k−2) I(k) I(k−1) I(k−2)], $\vartheta \left(k\right)=\left[b_{1}\;b_{2}\;b_{3}\;b_{4}\;b_{5}\right]$ and the sensor sampling error at moment *k* is e(k), U(k) can be expressed as:

(8) $$U\left(k\right)=\psi^{Τ}\left(k\right)\vartheta \left(k\right)+e\left(k\right)$$

The recursive least squares formula with the forgetting factor is as follows:

(9) $$\{\begin{array}{c} K\left(k\right)=\frac{P\left(k-1\right)\psi \left(k\right)}{\lambda +\psi^{T}\left(k\right)P\left(k-1\right)\psi \left(k\right)}\\ P\left(k\right)=\lambda^{-1}P\left(k-1\right)\left[I-K\left(k\right)\psi^{T}\left(k\right)\right]\\ \hat{\vartheta }\left(k\right)=\hat{\vartheta }\left(k-1\right)+K\left(k\right)\left[U\left(k\right)-\psi^{T}\left(k\right)\hat{\vartheta }\left(k-1\right)\right] \end{array}$$

where ψ is the error covariance matrix of FFRLS, K is the gain of FFRLS, and λ is the forgetting factor, generally 0<λ<1, where λ=0.95 is taken.

The relationship between the battery model parameters and coefficients is:

(10) $$\left\{ \begin{array}{c} R_{0}=\frac{b_{3}-b_{4}+b_{5}}{1+b_{1}-b_{2}}\\ \tau_{1}\tau_{2}=\frac{T^{2}\left(1+b_{1}-b_{2}\right)}{4\left(1-b_{1}-b_{2}\right)}\\ \tau_{1}+\tau_{2}=\frac{T\left(1+b_{2}\right)}{\left(1-b_{1}-b_{2}\right)}\\ R_{0}\tau_{1}+R_{0}\tau_{2}+R_{1}\tau_{2}+R_{2}\tau_{1}=\frac{T\left(b_{3}-b_{5}\right)}{\left(1-b_{1}-b_{2}\right)}\\ R_{0}+R_{1}+R_{2}=\frac{b_{3}+b_{4}+b_{5}}{\left(1-b_{1}-b_{2}\right)} \end{array}\right.$$

**Figure 2:**
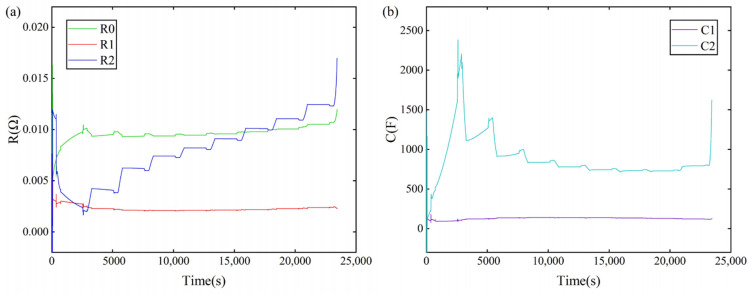
(**a**) $R_{0}$, $R_{1}$, $R_{2}$ parameter identification results; (**b**) $C_{1}$, $C_{2}$ parameter identification results.

The hybrid pulse power characterization (HPPC) experimental data are used for parameter identification, and the parameter identification results are shown in Figure 2.

## 3. SOC Estimation Based on AIEKF

In this paper, an AIEKF based on the Sage–Husa maximum a posteriori estimator is proposed for battery SOC estimation. The EKF linearizes the nonlinear system by truncating the higher-order terms through Taylor series expansion. Although the EKF improves the algorithm’s ability to handle nonlinear systems and simplifies the computational process, the resulting higher-order loss errors lead to a decrease in estimation accuracy. In order to solve the above problems in EKF, some optimization improvements are made to the EKF algorithm to reduce the influence of the above factors. The iterative idea is introduced in which the posterior estimate is substituted into the observation equation and the Jacobi matrix of the observation equation is updated by means of a multiple iterative measurement update process. By repeatedly using the observations, the estimates are continuously approximated to the true values during each iteration of the calculation. The absolute value of the difference between the measured end voltage and the estimated end voltage is used as the threshold value, and when the voltage error value is less than the threshold value, the iterative process is not required to reduce the computational burden. When the voltage error is greater than the threshold value, an iterative process is required. Because the system in the implementation of the estimation process will be affected by interference and random parameters, the process noise covariance and measurement noise covariance are constantly changing, which usually produces large estimation errors and affects the accuracy and robustness of SOC estimation. Incorporating the Sage–Husa maximum posteriori estimator and adaptively updating the process noise covariance matrix and measurement noise covariance matrix in real-time can improve the estimation accuracy and convergence speed of the algorithm under unknown system noise conditions, and further improve the filtering effect of the algorithm.

The nonlinear system model can be described as:

(11) $$\{\begin{array}{c} x_{k}=f\left(x_{k-1},u_{k-1}\right)+w_{k}\\ y_{k}=g\left(x_{k},u_{k}\right)+v_{k} \end{array}$$

Initialization:

(12) $$\{\begin{array}{c} {\hat{x}}_{0}=E\left(x_{0}\right)\\ P_{0}=E\left[\left(x_{0}-{\hat{x}}_{0}\right){\left(x_{0}-{\hat{x}}_{0}\right)}^{T}\right] \end{array}$$

where ${\hat{x}}_{0}$ is the initial estimate and $P_{0}$ is the initial covariance matrix.

Time to update:

(13) $${\hat{x}}_{k|k-1}=f\left(x_{k-1|k-1},u_{k-1}\right)$$

(14) $${\hat{P}}_{k|k-1}=A_{k}P_{k-1|k-1}A_{k}^{T}+Q_{k}$$

where $A_{K}={\frac{\partial f}{\partial \left(x\right)}|}_{x_{k}={\hat{x}}_{k|k-1}}$.

Measurement update:

Let the number of iterations be N, the estimated value of the ith iteration be $x_{k|k}^{i}$, and the estimated covariance be $P_{k|k}^{i}$

(15) $$x_{k|k}^{i}={\hat{x}}_{k|k-1}^{i}+K_{k}^{i}\left(y_{k}-{\hat{y}}_{k}^{i}\right)$$

(16) $$K_{k}^{i}={\hat{P}}_{k|k-1}^{i}C_{k}^{i}{\left(C_{k}^{i}{\hat{P}}_{k|k-1}^{i}C_{k}^{i}+R_{k}\right)}^{-1}$$

(17) $$P_{k|k}^{i}={\hat{P}}_{k|k-1}^{i}-K_{k}^{i}C_{k}^{i}{\hat{P}}_{k|k-1}^{i}$$

where $C_{k}^{i}={\frac{\partial g}{\partial \left(x\right)}|}_{x_{k}={\hat{x}}_{k}^{i}}$.

The absolute value of the difference between the measured terminal voltage and the estimated terminal voltage is used as the threshold value, and the threshold value is set to σ. When $\Delta_{k}=\left|y_{k}-{\hat{y}}_{k}\right|<\sigma$, no iterative process is required. When $\Delta_{k}=\left|y_{k}-{\hat{y}}_{k}\right|>\sigma$, the iterative process is performed.

Adaptive update:

Adaptive correction for process noise and measurement noise using an improved Sage–Husa estimator.

(18) $$e_{k}=y_{k}-g\left(x_{k},u_{k}\right)$$

(19) $${\hat{R}}_{k}=\left(1-d_{k}\right){\hat{R}}_{k-1}+d_{k}e_{k}e_{k}^{T}-C_{k}P_{k}C_{k}^{T}$$

(20) $${\hat{Q}}_{k}=\left(1-d_{k}\right){\hat{Q}}_{k-1}+\left(K_{k}e_{k}e_{k}^{T}K_{K}^{T}+P_{k}-A_{k|k-1}P_{k-1}A_{k|k-1}^{T}\right)$$

where $d_{k}=\frac{1-b}{1-b^{k+1}}$, and b is the forgetting factor.

The method uses the measurement data for recursive filtering while estimating and correcting the statistical characteristics of the system process noise and measurement noise in real-time, thus achieving the purpose of adaptive filtering.

## 4. Experimental Results and Discussion

### 4.1. Battery Test Platform

**Figure 3:**
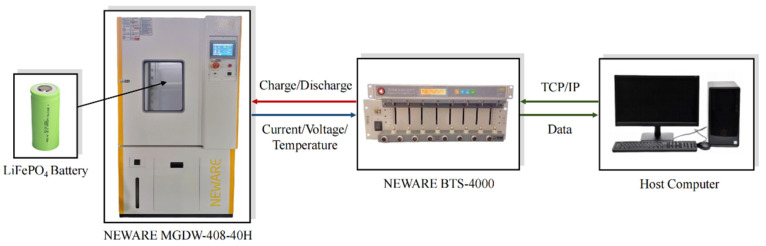
Battery test platform configuration.

In order to obtain the experimental data of battery current, voltage and capacity, we set up an experimental platform consisting of the host computer and the NEWARE BTS-4000 battery charge/discharge test system for experimental verification, whose structural configuration is shown in Figure 3. The host computer was connected to the battery test equipment for online control and data collection, and the NEWARE BTS-4000H was used to load the battery voltage and current. In this paper, a LiFePO_4_ battery with a nominal capacity of 6 Ah was selected as the experimental test object.

### 4.2. SOC–OCV–T Test

**Figure 4:**
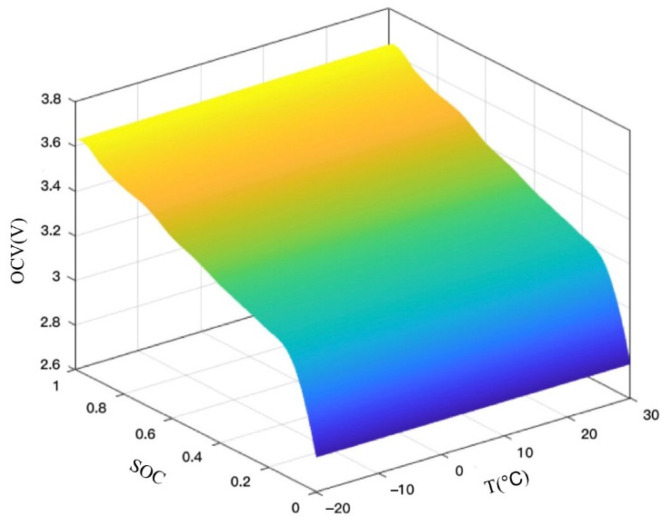
Experimental results of SOC–OCV–T at different temperatures.

**Figure 5:**
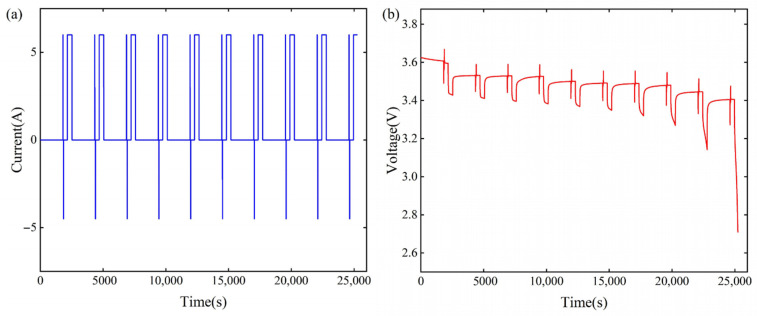
(**a**) HPPC operating condition current; (**b**) HPPC operating condition terminal voltage.

The discharge characteristics of the battery were experimented with by the HPPC test method [30,31]. The terminal voltage OCV of the battery in the operating state depends on the charge state SOC, so the open-circuit voltage at different SOCs needs to be collected in real-time. Because the SOC–OCV relationship is affected by temperature, the tests were performed in different temperature conditions. The effects of different temperatures on the SOC–OCV curves are shown in Figure 4. The HPPC operating current and cell terminal voltage at 25 °C are shown in Figure 5.

### 4.3. Analysis of Simulation and Experimental Results

**Figure 6:**
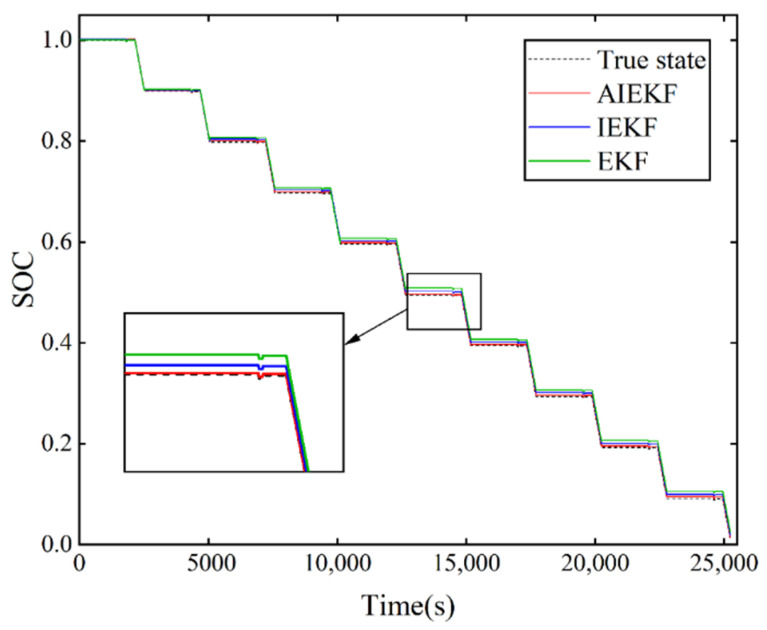
SOC estimation results of AIEKF, IEKF, and EKF under HPPC operating conditions.

**Figure 7:**
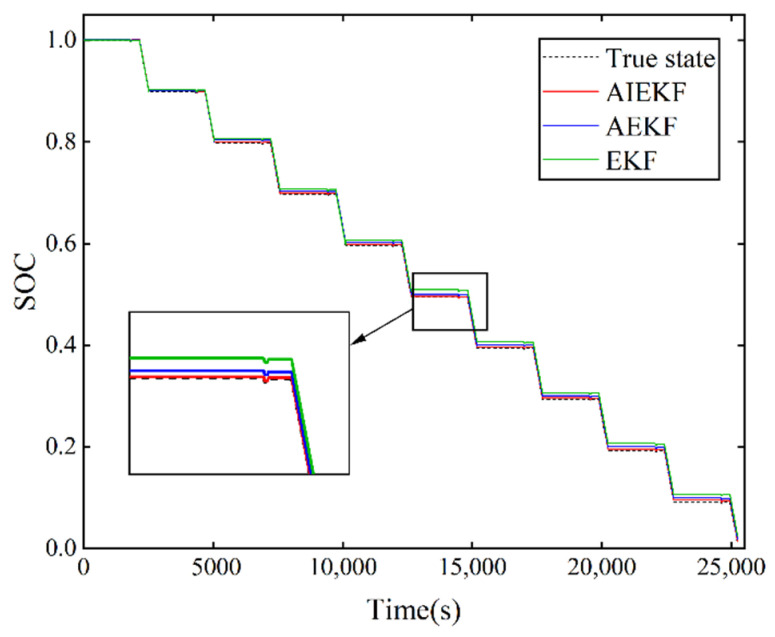
SOC estimation results of AIEKF, AEKF, and EKF under HPPC operating conditions.

In order to verify the effectiveness and superiority of the proposed method in terms of estimation accuracy, the proposed AIEKF is compared with the AEKF, IEKF, and EKF in this paper. The SOC estimation of the lithium-ion battery was performed under HPPC operating conditions and dynamic stress test (DST) operating conditions, and the estimated results are shown in Figure 6 and Figure 7. The actual SOC of the lithium-ion battery is obtained from Equation (2).

In this paper, the maximum estimation error (MAE) and the root mean square error (RMSE) are used as performance indicators to evaluate SOC estimation. The MAE and RMSE are calculated using Equations (21) and (22). The results of the MAE and RMSE calculations for the two operating conditions are shown in Table 1.

(21) $$MAE=max\left|SOC\left(t\right)-S\hat{O}C\left(t\right)\right|$$

(22) $$RMSE=\sqrt{\frac{1}{n}\overset{n}{\underset{k=1}{\sum }}{\left(SOC\left(t\right)-S\hat{O}C\left(t\right)\right)}^{2}}$$

where SOC(t) is the measured value at moment *t*, $S\hat{O}C\left(t\right)$ is the estimated value at moment *t*, and n is the number of data points in the sequence.

The performance of the lithium-ion battery under operating conditions is analyzed under HPPC operating conditions. Figure 6 shows the comparison results of SOC estimation for AIEKF, IEKF and EKF under HPPC operating condition. Figure 7 shows the comparison results of SOC estimation for AIEKF, AEKF and EKF. From Figure 6 and Figure 7, it can be seen that the SOC estimation results of AIEKF are closer to the real value, which is better than the other algorithms compared in this paper and has better estimation accuracy.

**Figure 8:**
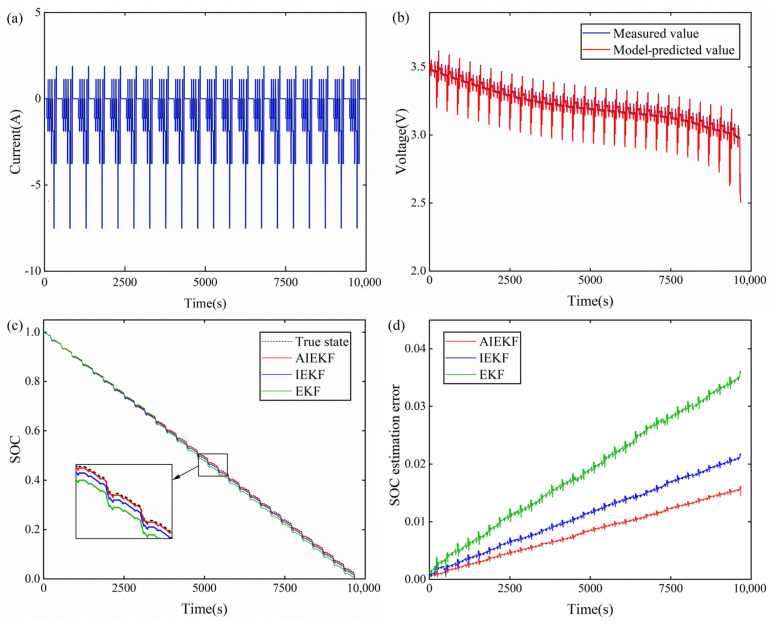
SOC estimation results of AIEKF, IEKF, and EKF under DST operating conditions: (**a**) current condition; (**b**) the model-predicted and the measured voltage; (**c**) SOC estimation results; (**d**) SOC estimation errors.

In order to further verify the reliability of the proposed method, a more complex DST condition was used to experiment with and analyze the lithium-ion battery. The DST current condition is shown in Figure 8a. The model-predicted voltage and the measured voltage are shown in Figure 8b. Figure 8c compares the SOC estimation results of AIEKF, IEKF, and EKF. Figure 8d compares the SOC estimation errors of the AIEKF, IEKF, and EKF. It can be seen from Figure 8 that the SOC estimation result of AIEKF is closer to the true value, and its estimated value continuously follows the true value. Additionally, the estimation error of AIEKF is smaller.

**Figure 9:**
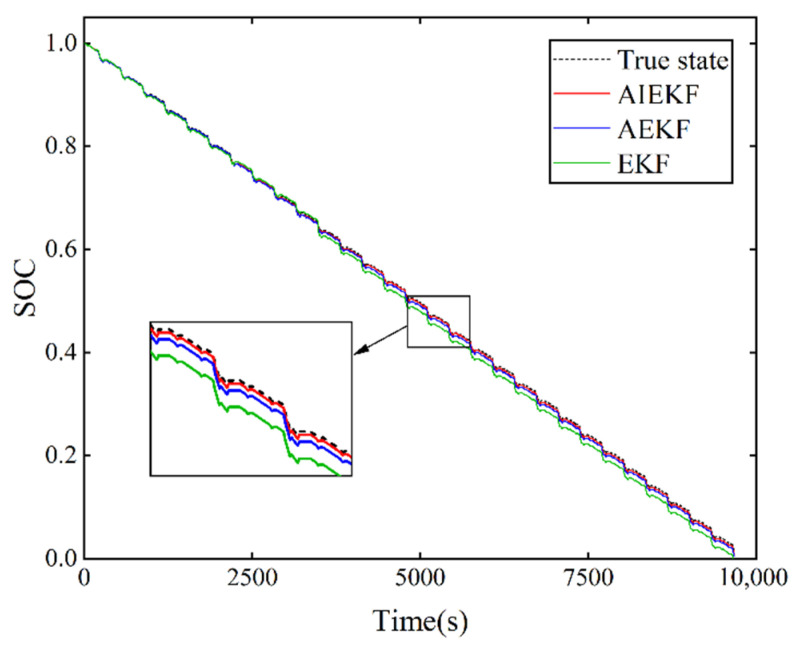
SOC estimation results of AIEKF, AEKF, and EKF under DST operating conditions.

Figure 9 shows the SOC estimation results of AIEKF, AEKF, and EKF. In Figure 9, we can see that the SOC estimation result of AIEKF is closer to the true value and the estimation results are better than AEKF, and then, we can verify that the SOC estimation accuracy can be further improved by introducing the iterative idea in the adaptive case.

The results show that the AIEKF-based SOC estimation method proposed in this paper can maintain lower SOC estimation error, higher tracking accuracy and lower fluctuation under two current conditions compared with the AEKF, IEKF and EKF. The MAE and RMSE of AIEKF were calculated as being less than 1%, and the data verify that the estimation accuracy of AIEKF was better than that of AEKF, IEKF and EKF, so AIEKF can effectively improve the SOC estimation accuracy. 

**Figure 10:**
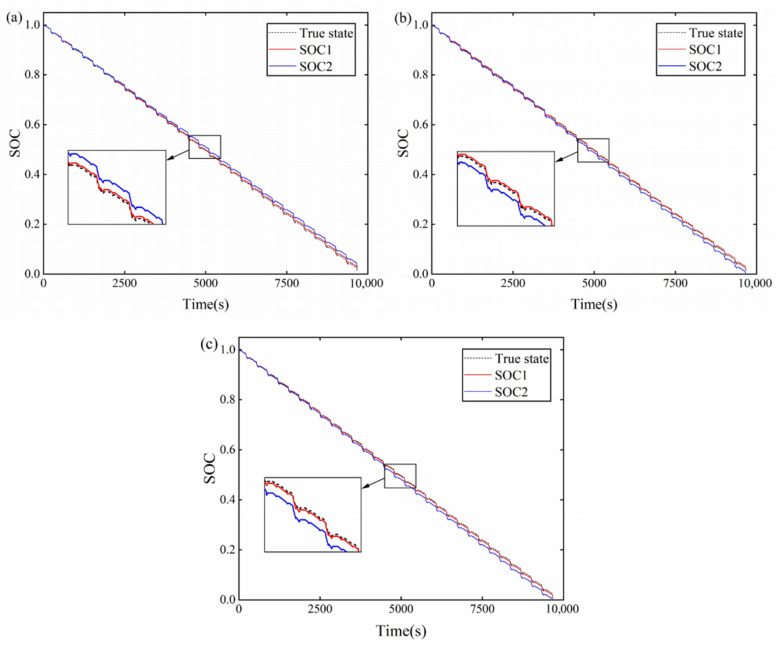
SOC estimation at (**a**) –20 °C; (**b**) 0 °C; (**c**) 30 °C.

Three temperatures of –20, 0, and 30 °C were selected to verify the estimation effect of the proposed method at different temperatures under DST operating conditions. The SOC estimation was performed at the three temperatures with and without considering the temperature effect, and the results are shown in Figure 10. In Figure 10, the SOC1 is the SOC estimation result with considering the temperature effect and the SOC2 is the SOC estimation result without considering the temperature effect. From Figure 10, it can be seen that the proposed AIEKF can efficiently perform SOC estimation at different temperatures in this paper.

By evaluating the SOC estimation performance of different KF family algorithms using model-based algorithms in previous studies with the proposed algorithm in this paper, the comparison results are shown in Table 2. The MAE < 1% of AIEKF is smaller among the results listed in Table 2, so the AIEKF proposed in this paper has a better estimation accuracy.

Although the AIEKF proposed in this paper can improve the accuracy of SOC estimation for lithium-ion batteries, the computational burden is still affected to some extent. The discrete-time model with a sampling period of 1 s was simulated using MATLAB/Simulink R2020b on a desktop with an Intel (R) Core (TM) i7-6500 CPU and 8 GB RAM. The computation times of the three estimation algorithms are listed in Table 3, and the evaluation demonstrates that the AIEKF proposed in this paper is able to reduce the unnecessary computational burden.

## 5. Conclusions

The SOC is a valuable parameter for the AUV’s control system to distribute electric energy to the propulsion system. In order to quickly obtain an accurate SOC, an improved AIEKF SOC estimation method for lithium-ion batteries was proposed in this paper. In addition, the FFRLS method was applied to update the parameters of the second-order RC equivalent circuit model of lithium-ion battery online to obtain an accurate model parameter. The computational accuracy was improved, and the unnecessary computational burden was reduced by setting a threshold value. An improved Sage–Husa estimator was used for an adaptive update optimization of process noise and measurement noise to eliminate the effect of noise. The validity of the AIEKF and its good estimation performance were demonstrated by experimental verification analysis with different currents and temperatures. The experimental results show that the RMSE of the SOC estimation results can reach 0.25%.

Further research would focus on the battery aging effect on SOC estimation and the prediction of the SOH of lithium-ion batteries in AUVs so that a more intelligent battery energy management system for AUVs can be established to adopt to a long-term voyage in the ocean environment.

## Figures and Tables

**Table 1:** Evaluation results of different SOC estimation methods under two operating conditions.

Method	HPPC		DST	
	MAE (%)	RMSE (%)	MAE (%)	RMSE (%)
EKF	1.2648	1.1783	1.4357	1.2518
IEKF	1.1450	0.9256	1.3274	1.0392
AEKF	0.9715	0.7482	1.1509	0.8253
AIEKF	0.6582	0.2549	0.8326	0.3471

**Table 2:** Comparison of different SOC estimation methods.

Reference	Method	Model	MAE (%)
[32]	EKF	Rint	<4
[33]	AEKF	2RC	<2
[34]	UKF	1RC	<1.7
[35]	AUKF	2RC	<1.5

**Table 3:** Computational burden evaluation.

Method	Computational Time (s)
EKF	0.6418
IEKF	1.1273
AEKF	1.0849
AIEKF	0.9524
